# Uncoupling DISC1 × D2R Protein-Protein Interactions Facilitates Latent Inhibition in Disc1-L100P Animal Model of Schizophrenia and Enhances Synaptic Plasticity via D2 Receptors

**DOI:** 10.3389/fnsyn.2018.00031

**Published:** 2018-09-07

**Authors:** Tatiana V. Lipina, Nikolay A. Beregovoy, Alina A. Tkachenko, Ekaterina S. Petrova, Marina V. Starostina, Qiang Zhou, Shupeng Li

**Affiliations:** ^1^Federal State Budgetary Scientific Institution, Scientific Research Institute of Physiology and Basic Medicine, Novosibirsk, Russia; ^2^Institute for the Medicine and Psychology of Novosibirsk State University, Novosibirsk, Russia; ^3^Institute of Molecular Biology and Biophysics, FRC FTM, Novosibirsk, Russia; ^4^School of Chemical Biology and Biotechnology, Shenzhen Graduate School, Peking University, Shenzhen, China

**Keywords:** DISC1, D2R, Disc1-L100P mouse model of schizophrenia, latent inhibition, synaptic plasticity

## Abstract

Both Disrupted-In-Schizophrenia-1 (DISC1) and dopamine receptors D2R have significant contributions to the pathogenesis of schizophrenia. Our previous study demonstrated that DISC1 binds to D2R and such protein-protein interaction is enhanced in patients with schizophrenia and Disc1-L100P mouse model of schizophrenia (Su et al., 2014). By uncoupling DISC1 × D2R interaction (trans-activator of transcription (TAT)-D2pep), the synthesized TAT-peptide elicited antipsychotic-like effects in pharmacological and genetic animal models, without motor side effects as tardive dyskinesia commonly seen with typical antipsychotic drugs (APDs), indicating that the potential of TAT-D2pep of becoming a new APD. Therefore, in the current study, we further explored the APD-associated capacities of TAT-D2pep. We found that TAT-D2pep corrected the disrupted latent inhibition (LI), as a hallmark of schizophrenia associated endophenotype, in Disc1-L100P mutant mice—a genetic model of schizophrenia, supporting further APD’ capacity of TAT-D2pep. Moreover, we found that TAT-D2pep elicited nootropic effects in C57BL/6NCrl inbred mice, suggesting that TAT-D2pep acts as a cognitive enhancer, a desirable feature of APDs of the new generation. Namely, TAT-D2pep improved working memory in T-maze, and cognitive flexibility assessed by the LI paradigm, in C57BL/6N mice. Next, we assessed the impact of TAT-D2pep on hippocampal long-term plasticity (LTP) under basal conditions and upon stimulation of D2 receptors using quinpirole. We found comparable effects of TAT-D2pep and its control TAT-D2pep-scrambled peptide (TAT-D2pep-sc) under basal conditions. However, under stimulation of D2R by quinpirole, LTP was enhanced in hippocampal slices incubated with TAT-D2pep, supporting the notion that TAT-D2pep acts in a dopamine-dependent manner and acts as synaptic enhancer. Overall, our experiments demonstrated implication of DISC1 × D2R protein-protein interactions into mechanisms of cognitive and synaptic plasticity, which help to further understand molecular-cellular mechanisms of APD of the next generation.

## Introduction

Schizophrenia is a chronic brain disorder associated with severe psychotic symptoms which lead to disability. The prevalence of schizophrenia remains stably high and currently near 21 million people suffer from this mental disorder, according to World Health Organization (World Health Statistics, 2017). Schizophrenia is characterized by negative (social withdrawal, motivational deficit), positive (delusions, hallucinations and bizarre thoughts) symptoms and cognitive symptoms, which are considered as core symptoms of this mental illness (Elvevåg and Goldberg, 2000) since they are observed among all subtypes of schizophrenia (Heinrichs and Awad, 1993). However, there are no effective treatments of cognitive impairments in schizophrenics as typical and atypical antipsychotic drugs (APDs) only partly ameliorate the positive or negative symptoms of schizophrenia (Meltzer, 1995) and near 60% of schizophrenics are resistant to APDs treatment (Meltzer and Kostacoglu, 2001). Moreover, currently used APDs often cause side-effects, including extrapyramidal syndrome, gain weight, dyskinesia, diabetes and dysfunction of reproductive system (Leucht et al., 2009).

D2 dopamine receptor (D2R) is a classical target of current APDs and most effective APDs antagonize the D2R (Seeman and Kapur, 2000; Glatt et al., 2003). Clinical efficacy of APDs positively correlates with their binding capacity to D2R (Seeman et al., 1976). Additionally, D2R mRNA and protein expression levels are elevated in the brain of patients with schizophrenia as shown in post-mortem, PET and SPECT studies (Roberts et al., 1994; Seeman and Kapur, 2000). There is a need for a deeper understanding of the D2R signaling pathways in order to reveal new therapeutic targets for more effective APDs with reduced side effects.

D2R belong to a family of G-protein coupled receptors mainly mediating G-protein dependent pathways (Missale et al., 1998). D2R elicits its action through intracellular Gi/Go proteins, which control adenylate cyclase enzymatic activity, exchange of phosphatidylinositol, release of arachidonic acid, activities of K^+^ and Ca^2+^ ionic channels and protein kinases (Missale et al., 1998). D2R may also act independently on G-proteins, e.g., via β-arrestin-2, which initiates the formation of a complex with protein phosphatase-2A (PP2A), protein kinase B (PKB or Akt), activation of glycogen synthase kinase-3 (GSK-3; Beaulieu et al., 2009). The main mechanism for regulating D2R is kinase-dependent desensitization of D2R, endocytosis and endosomal trafficking, which then leads to the formation of a complex with β-arrestin-2, adaptor protein-2 (AP2) and clathrin (Hanyaloglu and von Zastrow, 2008).

Studies of D2R interacting proteins, using yeast two-hybrid, co-immunoprecipitation, glutathione S-transferase (GST) pull-down and *in vitro* binding assays, have identified about 20 proteins with many of them relevant to schizophrenia (Wang et al., 2008; Shioda et al., 2010; Kabbani et al., 2012). These proteins selectively regulate specific signaling pathways and function of D2R via protein-protein interactions, without affecting other signaling pathways. Thus, targeting protein-protein interactions may represent a promising alternative approach to treat schizophrenia, which might eliminate side effects of current APDs.

Indeed, synthesized peptides that mimic the binding domain responsible for D2R-protein interactions interfere with D2R-protein interactions and can effectively disrupt such D2R-protein complexes to selectively block certain signaling pathway. The trans-activator of transcription (TAT) domain of the human immunodeficiency virus (HIV) can be fused to these small peptides to facilitate their intracellular entry to initiates the action of these peptides. There are accumulating studies shown various effects of TAT-peptides affecting D2R-peptide interactions on function of the central nervous system (CNS; Su et al., 2015). For instance, administration of TAT-peptide that affects D2R × dopamine transporter (DAT) interaction in mice decreased dopamine uptake and increased their ambulation in a similar way as in the DAT knockout mice (Lee et al., 2007). D2Rs also directly interact with the C-terminus of the GluN2B subunit of N-Methyl-D-aspartate (NMDA) ionotropic glutamate receptors through an N-terminal 10 amino acid motif of its third cytoplasmic domain (Liu et al., 2006). Cocaine enhances this interaction in the striatum and treatment with TAT-tagged peptide that encodes the interacting site of the D2R-NR2B interaction, inhibits cocaine-induced locomotion, highlighting another dopamine-glutamate system interaction that may be a useful target for treating addiction (Liu et al., 2006). Another example of affecting the function of the CNS via TAT-peptides affecting D2R protein-protein interactions is the peptide interfering with interaction of D2R with neuronal calcium sensor-1 (NCS-1) which showed decreased explorative behavior, hippocampal long-term potentiation (LTP) and spatial memory in mice (Saab et al., 2009). NCS-1 regulates the phosphorylation, trafficking and signaling transduction of D2R in neurons. NCS-1 may contribute to the molecular mechanisms of schizophrenia and might be involved in the action of APDs (Koh et al., 2003; Bai et al., 2004). Our recent study (Su et al., 2014) found that Disrupted-In-Schizophrenia-1 (DISC1), one of robust gene-candidate associated with schizophrenia (Brandon and Sawa, 2011; Porteous et al., 2011) interacts with D2R through the third intracellular loop of D2R. The levels of DISC1 × D2R protein complex is increased in the postmortem brain tissues of schizophrenics and Disc1-L100P mutant mice, a genetic model of schizophrenia (Clapcote et al., 2007; Lipina and Roder, 2014). DISC1 × D2R interactions facilitated D2R-mediated GSK-3 signaling and inhibited D2R internalization, supporting hyperactivity of dopaminergic system which was observed in patients with schizophrenia (Davis et al., 1991). TAT-peptide uncoupling DISC1 × D2R complex reversed hyperactivity and deficit of sensorimotor gaiting, induced by either amphetamine/apomorphine in rats or by genetic Disc1-L100P mutation in mice (Su et al., 2014), hence, eliciting APDs features in both pharmacological and genetic animal models of schizophrenia. Notably, pharmacological inhibitors of other DISC1 interacting proteins, such as GSK-3 (Lipina et al., 2011a) and PDE4B (Clapcote et al., 2007), also induced APD-like capacities in Disc1-L100P mice and dual inhibitor of GSK-3 and PDE7, VP1.15, elicited APDs features in amphetamine pharmacological model of schizophrenia (Lipina et al., 2013).

Given that TAT-D2R peptide uncoupling DISC1 × D2R protein-protein interactions exhibited APD-related capacities without side effects (Su et al., 2014), we aimed in the current study to further explore APD-related features of TAT-D2R peptide (TAT-D2pep). Animal assays associated with schizophrenia represent an important preclinical tool for testing novel pharmacological compounds in the treatment of schizophrenia. Disc1-L100P mutant mice exhibit several schizophrenia-related behavioral phenotypes, including hyperactivity, deficits of sensorimotor gaiting, assessed by pre-pulse inhibition (PPI) of acoustic startle response, working memory and latent inhibition (LI) of fear conditioning (Clapcote et al., 2007). LI is among important behavioral models in neuropharmacological research of schizophrenia with face, predictive and construct validity (Geyer and Ellenbroek, 2003). LI reflects an organism’s ability to ignore irrelevant stimuli (Gray et al., 1992). LI paradigms are usually based on between-subject design and consist of one group of subjects being pre-exposed (PE) to a neutral to-be-conditioning stimulus (CS), whereas another group of subjects are not pre-exposed (NPE). The CS is subsequently paired with an unconditioned stimulus (US). LI is measured by the difference to learn the CS–US association between the PE and NPE groups and consists of a retardation of learning in the PE group. Healthy humans or rodents treated with amphetamine show the disrupted LI (Weiner et al., 1984; Gray et al., 1992; Lipina and Roder, 2010) and in the acute stages of schizophrenia (Weiner et al., 1984, 1988; Gray et al., 1992; Thornton et al., 1996; Rascle et al., 2001). Both typical and atypical APDs reverse amphetamine-induced disruption of LI and reliably potentiate LI under conditions that normally do not yield robust LI (Weiner and Feldon, 1997; Moser et al., 2000). We showed that clozapine, GSK-3 and PDE4 inhibitors corrected the disrupted LI in Disc1-L100P mice (Clapcote et al., 2007; Lipina et al., 2011a). TAT-D2pep was able to improve hyperactivity and PPI deficit in Disc1-L100P mice (Su et al., 2014), however, it remains to be explored if LI deficit could be rescued by uncoupling Disc1 × D2R interactions in Disc1-L100P genetic model of schizophrenia.

Of particular interest was also to probe the efficacy of TAT-D2pep as a cognitive enhancer. Indeed, a special program, Cognitive Neuroscience measures of Treatment Response of Impaired Cognition in Schizophrenia (CNTRICS) was developed, which suggests that screening of novel APDs for their capacities as cognitive enhancers by incorporating a variety of cognitive behavioral tests, suitable genetic mouse models and parametric manipulations of the behavioral models (Barak and Weiner, 2011). Since the main aim in animal research on cognitive enhancement is to develop compounds to ameliorate cognitive deficits associated with e.g., schizophrenia, then it should be asked how these drugs may affect cognition in normal individuals. For instance, typical antipsychotics, sulpiride or clozapine ameliorated attentional deficit or working memory deficit in animals with disrupted prefrontal cortical functions but these antipsychotics impaired performance of control animals (Murphy et al., 1997; Passetti et al., 2003a,b; Baviera et al., 2008). Hence, there is an important discussion in the field whether interventions that will improve cognition in patients with mental disorders can be also successfully detected in healthy subjects. Therefore, the second goal of the current study was to probe if TAT-D2pep can facilitate cognitive functions in control inbred mouse strain C57BL/6NCrl and examine whether TAT-D2pep affects synaptic plasticity to underlie its potential cognitive enhancing capacities.

## Materials and Methods

### Animals

Disc1-L100P homozygous male mice and their wild-type (WT) littermates, C57BL/6NCrl (C57BL/6N) inbred strain were bred in the animal facility of Scientific Research Institute of Physiology and Basic Medicine (SRIPhBM). Experiments were conducted on 2–3 months old Disc1-L100P\WT mice (Experiment 1) and C57BL/6N mice (Experiments 2–3). Homozygous Disc1-L100P male mice and WT mice (DISC1-L100P^+/+^ or C57BL/6NCrl; WT) were obtained from breeding of heterozygous pairs. Genotyping was performed with mild modifications as described before Clapcote et al. (2007), using primers: F, 5′-CCACTGCCAAGCCTCACT-3′ and R: 5′-GGCCACAGCAGGGACAA-3′. All mice were housed five per cage in the vivarium of the SRIPhBM in plastic cages (OptiMice Biotech AS, 34 × 29 × 15 cm) in a temperature-controlled room (21–23°C) with a reversed light-dark cycle (12 h/12 h; lights on: 18:00 h; lights off: 6:00 h) with food and water available *ad libitum*. This study was conducted in accordance with the recommendations of European Communities Council Directive of September 22, 2010 (2010/63/EU; Ethics Committee of the SRIPhBM). All experimental protocols were approved by the Ethics Committee of the SRIPhBM.

### Behavioral Studies

Behavioral tests were done between 9 am and 4 pm. Prior to all experiments mice were acclimatized to the experimental room for 30 min. The behavioral equipment was cleaned with 70% ethanol between mice to remove residual odors.

### Latent Inhibition (LI) in the Conditioned Freezing Paradigm

The LI in the conditioning freezing paradigm was performed according to previously published protocol (Yee et al., 2006). The experiment was conducted in a fear conditioning apparatus (Med Associates, St. Albans, VT, USA) consisting of a test chamber (25 cm high × 30 cm wide × 25 cm deep) equipped with a computer-controlled fear conditioning system (Actimetrics, Wilmette, IL, USA). Freezing behavior was recorded using automated fear conditioning software (Actimetrics software; FREEZFRAME v 1.6e) during the four phases of the procedure as follows: *pre-exposure*, *conditioning* (day 1), and *tone test* (day 2). Pre-exposure and conditioning were conducted consecutively, on the same day of the experiment. The peptide was injected 30 min before the behavioral procedure on the 1st day. The PE animals received 40 presentations of a 30 s tone CS (80 dB, a 3.6 kHz pulsated tone) at a variable interstimulus interval of 40 ± 30 s; non-pre-exposed (NPE) mice were confined to the chamber for an equivalent period of time. Conditioning commenced immediately at the end of pre-exposure without removing the animals from the chambers. Conditioning comprised either two discrete trials of CS-US pairing (Experiment 1) or five CS-US pairs in order to detect cognitive facilitation in C57BL/6N mice (Experiment 2). Each trial began with the 30 s tone stimulus (the same as the one used during pre-exposure) followed immediately by the delivery of a 1 s foot shock set at 0.4 mA with 180 s interval between CS-US trials. On the tone test (24 h after the conditioning), the context was altered and each mouse was placed into the altered chamber and allowed 180 s for exploration (pre-tone freezing), after which the auditory tone cue was turned on for 300 s. The citrus scent was used on the 1st day and vanilla scent on the 2nd day as additional environmental cues. Data are presented as percentage of freezing every 60 s during the tone test. Percentage of the averaged freezing was calculated based on the freezing in response to the CS.

### T-Maze

T-maze was performed as described before (Lipina et al., 2013). In this test, the animal alternates between two goal arms during repetitive visits based on the recall of the previously visited arm. The T-maze apparatus is made of gray Plexiglas with a main stem (65 × 14 × 30 cm) and two arms (30 × 14 × 30 cm) positioned at a 90° angle relative to the left and right of the main stem. A start box (10 × 14 × 30 cm) is separated from the main stem by a sliding door. The experimental protocol consists of one single session, which starts with trial, when the animal is confined for 5 s in the start box and then released while both the left and right arms were not blocked by the sliding door and the mouse is free to choose between the left, right arms and the main stem. The animal is considered as entered when it places its four paws in the arm. A session is terminated and the animal is removed from the maze after 5 min. Spontaneous alternation is defined as entry in a different arm of the T-maze over successive trials (i.e., ABC, CBA). The percentage of alterations was defined according to the following equation: % Alteration = [(Number of alterations)/(Total arm entries − 2)]*100. The number of arm entries serves as an indicator of ambulation.

### Electrophysiological Studies

Transverse hippocampal slices (400 μm thick) were prepared from the brains of C57BL/6N inbred adult male mice. Mice were sacrificed by cervical dislocation, brains were rapidly removed and chilled in ice-cold Ca^2+^-free solution, and slices were prepared in ice-cold Ca^2+^-free cutting solution (Mathis et al., 2011) using a vibroslicer (NVSL, World Precision Instruments). Cutting solution composition included: 124 mM NaCl, 2 mM KCl, 1.25 mM KH_2_PO_4_, 2 mM MgSO_4_, 26 mM NaHCO_3_ and 10 mM dextrose, pH 7, 4.

Experiments were conducted as described previously (Beregovoy et al., 2011). Briefly, the slices were allowed to recover for at least 1 h before an experiments in artificial CSF (ACSF), saturated with 95% O_2_ and 5% CO_2_ at temperature 34°C and were then transferred to an experimental chamber for extracellular recordings. The flow rate of the solution through the chamber was 1 ml/min. The composition of the ACSF was 124 mM NaCl, 2 mM KCl, 26 mM NaHCO_3_, 1.25 mM KH_2_PO_4_, 2.5 mM CaCl_2_, 2 mM MgSO_4_ and 10 mM dextrose, bubbled with a 95% O_2_–5% CO_2_ mixture, and had a final pH of 7.4. All experiments were performed at room temperature. Quinpirole (Sigma) or peptides (TAT-D2pep or TAT-D2pep-sc) were added into experimental solution in final concentration 10 μM.

Extracellular recordings of field excitatory postsynaptic potentials (fEPSPs) were obtained from the stratum radiatum of the CA1 region of the hippocampus using glass micropipettes filled with ACSF. The stimulating electrode was a bipolar concentric electrode, and the recording electrode was 1.5 mm borosilicate glass (Institute for Biological Instrumentation of the Russian Academy of Sciences, IBI RAS) containing ACSF with a resistance of about 2–4 MΩ. Stimulus was applied to the Schaffer collateral via a concentric bipolar stainless steel electrode (A 360, World Precision Instruments). Stimulus pulses consisted of a single square wave of 100 μs duration delivered at 30–150 μA.

To determine the test stimulus intensity, a paired-pulse test was done, and the stimulus intensity was adjusted to the point at which a population spike was evoked on the second, but not the first, pulse as shown in Figure 3A. This protocol resulted in a stimulus that was 30%–50% of the intensity required to elicit a maximum response (Salazar-Weber and Smith, 2011). Subsequent fEPSPs were elicited once per 1 or 2 min at this stimulation intensity. High frequency stimulation (HFS or tetanization; three 1 s trains at 100 Hz, intertrain interval 20 s) was used to induce LTP. Signals low pass filtered at 1 kHz, digitized with a Digidata 1200 A/D converter at 10 kHz, and analyzed with pCLAMP software (Axon Instruments). Data was analyzed offline using pClamp and Microcal Origin 8.1 software (OriginLab). Amplitudes of fEPSPs were normalized with respect to the 10-min control period before drug application or HFS.

**Figure 1:**
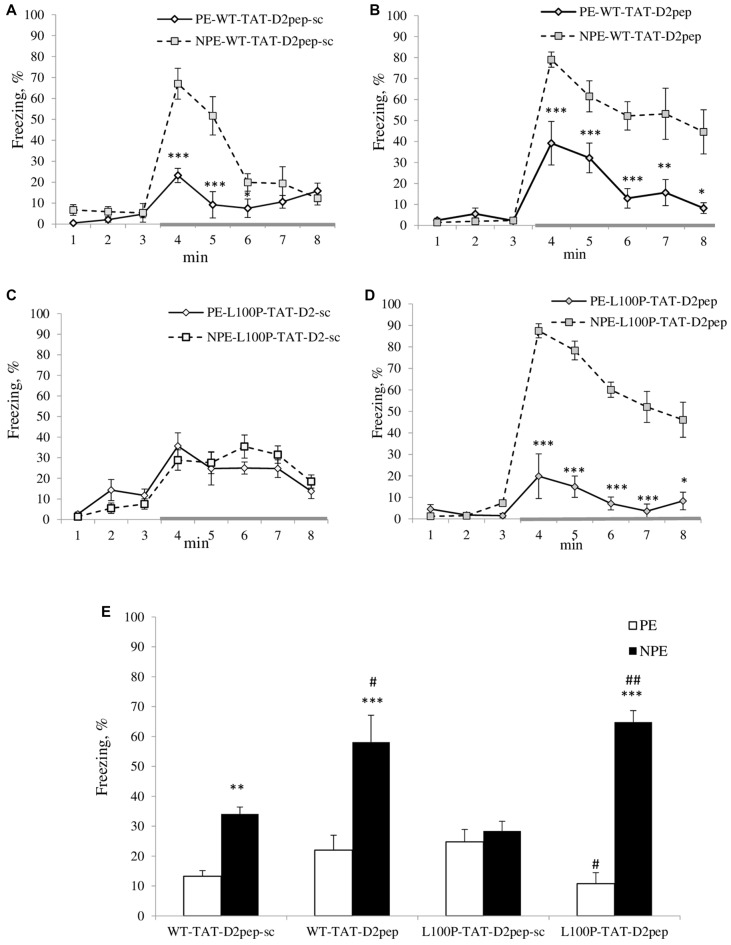
**(A–E)** Trans-activator of transcription (TAT)-D2pep reversed deficient latent inhibition (LI) in disrupted-in-schizophrenia-1 (Disc1)-L100P mice. **(A)** TAT-D2pep-sc (3 nmol/kg; i.p.; 30 min) did not affect LI in wild-type (WT) mice. **(B)** TAT-D2pep increased the duration of the freezing in response to the tone (conditioned stimulus, CS; gray colored line) in WT mice. **(C)** TAT-D2pep-sc had no effect on the disrupted LI in Disc1-L100P mice. **(D)** Uncoupling DISC1 × D2R by the peptide significantly improved expression of LI in Disc1-L100P mice. **(E)** Percentage of the averaged freezing in response to the CS for each experimental group. Saline-treated mice did not differ from TAT-D2pep-treated animals (data not shown). *N* = 7–8 mice per group; *p < 0.05; **p < 0.01; ***p < 0.001—in comparison with non pre-exposed (NPE) group within each treatment. ^#^*p* < 0.05; ^##^*p* < 0.01—in comparison with TAT-D2pep-sc-treated pre-exposed (PE)\NPE groups within each genotype.

**Figure 2:**
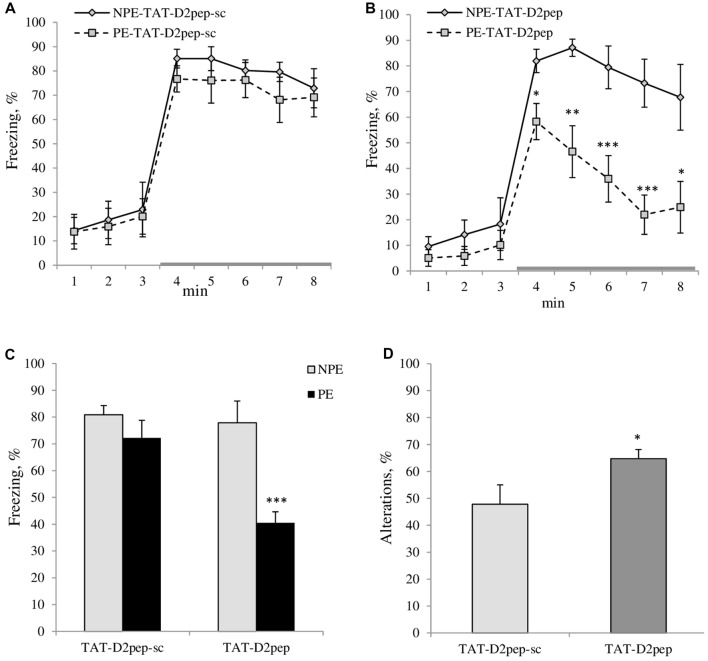
**(A–C)** TAT-D2pep facilitated working memory and LI in C57BL/6N mice. **(A)** LI is disrupted by five CS-US pairings in TAT-D2pep-sc-treated C57Bl/6N mice since the PE to the tone (CS; PE) and NPE to the tone (NPE) groups of experimental mice expressed comparable freezing (%) in response to the CS (gray colored line), whereas **(B)** TAT-D2-pep-treated PE mice significantly reduced their freezing in response to the CS as compared with their NPE group, demonstrating LI under conditions with five CS-US pairings. **(C)** Percentage of the averaged freezing in response to the CS for each experimental group. Saline-treated mice did not differ from TAT-D2pep-treated animals (data not shown). *N* = 6–8 mice per group; *p < 0.05; **p < 0.01; ***p < 0.001—in comparison with NPE group. **(D)** TAT-D2pep (3 nmol/kg; i.p.; 30 min) increased the number of spontaneous alterations (%), assessed in T-maze. *N* = 8 mice per group; *p < 0.05—in comparison with TAT-D2pep-sc-treated animals.

**Figure 3:**
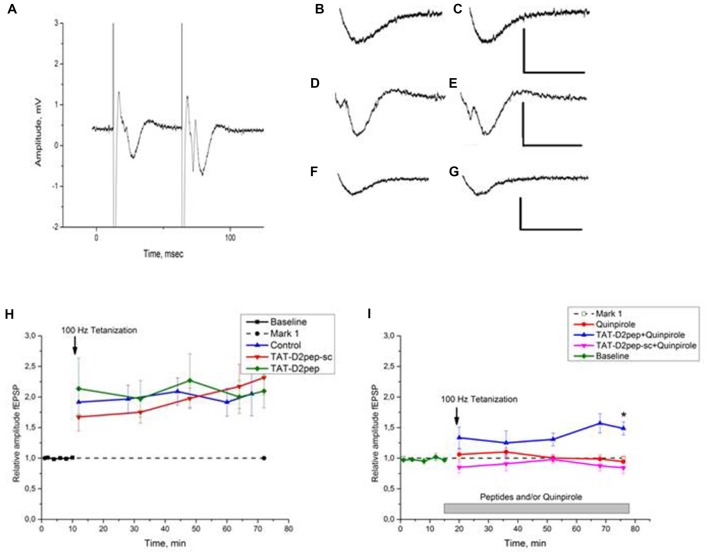
**(A–I)**. Effects of TAT-D2pep on synaptic plasticity of the mouse hippocampal CA1 region.** (A)** Typical recordings from the mouse hippocampal slice. A representative trace from the CA1 stratum radiatum (paired-pulse test at the stimulus intensity chosen for recording). Interstimulus interval 50 ms. Stimulating bipolar concentric electrode in the Schaffer collateral. **(B–G)** *Effects of TAT-D2pep-sc, TAT-D2pep and quinpirole on baseline fEPSP responses.* Scale: 1 mV, 20 ms; **(B–C)** representative traces responses before **(B)** and after **(C)** 10 min incubation with 10 μM TAT-D2pep-sc, respectively; **(D–E)** fEPSP before **(D)** and after **(E)** 10 min incubation with 10 μM TAT-D2pep; **(F–G)** fEPSP before **(F)** and after **(G)** 10 min incubation with 10 μM quinpirole, respectively. **(H)** *Effects of TAT-D2pep, TAT-D2pep-sc on fEPSP amplitude in CA1 hippocampal region.* Long term potentiation (LTP) was recorded for 2 min, 18 min, 34 min, 50 and 58 min after the stimulation. Baseline: 0–10 min. Control—saline application. Ordinate: relative amplitude fEPSP. The normalized fEPSP amplitude is shown in the presence of 10 μM TAT-D2pep or 10 μM TAT-D2pep-sc before and after application of High frequency stimulation (HFS) protocol (arrow). **(I)** *Effects of TAT-D2pep, TAT-D2pep-sc and Quinpirole on relative amplitude fEPSP in CA1.* The normalized fEPSP amplitude in the presence 10 μM Quinpirole, 10 μM TAT-D2pep or 10 μM TAT-D2pep-sc in different combination after application of HFS protocol (arrow). *N* = 6–7 mice per group, with 2–3 slices from each animal; *p < 0.05.

Drug application: Quinpirole (LY-1, 71, 555; Sigma), TAT-D2pep and TAT-D2pep-sc were dissolved in standard ACSF for the concentration of 10 μM for all compounds, used in the electrophysiological experiments. All experiments were monitored drug-free for at least 10 min to measure potential drug effects on baseline fEPSP responses. Experiments in which no drugs were used were then tetanized at *t* = 10 min. In experiments using one drug, it was added at *t* = 10 min; tetanization at *t* = 20 min. In experiments using two drugs, the first was added at *t* = 10 min, the second at *t* = 20 min with tetanization at *t* = 30 min. All drugs, once added, were maintained throughout the experiment.

### Peptide

Disc1 × D2R uncoupling peptide (TAT-D2pep) and its controlled compound—Disc1 × D2R scrambled peptide (TAT-D2pep-sc) were synthesized as previously described (Su et al., 2014). TAT-D2pep (Tyr-Gly-Arg-Lys-Lys-Arg-Arg-Gln-Arg-Arg-Arg-Lys-Ile-Tyr-Ile-Val-Leu-Arg-Arg-Arg-Arg-Lys-Arg-Val-Asn-Thr; molecular weight: 3513.3) and TAT-D2pep-sc (Tyr-Gly-Arg- Lys- Lys- Arg- Arg- Gln- Arg- Arg- Arg- Val- Leu- Arg- Lys-Thr- Arg-Ile- Arg- Arg-Tyr- Lys- Ile- Arg-Asn-Val molecular weight: 3511.2) were dissolved in 0.9% NaCl saline (3 nmol/g, i.p.) and administrated in a volume 10 ml/kg with 30 min as injection-testing interval in the 1st day of LI procedure.

### Statistics

Behavioral data were analyzed by ANOVA with genotype as a between-subject main factor and time-intervals as a repeated measure. Significant main effects and interactions were followed by LSD *post hoc* comparisons to assess the differences between experimental groups. Electrophysiological and cell culture data were analyzed using Microcal Origin (OriginLab, Northampton, MA, USA) software. Statistical significance was evaluated using one-way ANOVA to compare unpaired conditions. Paired samples between the control condition (before drug application or before HFS) and a given time after HFS were analyzed using the parametrical *t*-test.

### Experimental Design

#### Experiment 1

The efficacy of TAT-D2pep was probed to rescue LI deficit in Disc1-L100P mice in comparison with their WT littermates, using regular LI protocol with 2 CS-US.

#### Experiment 2

Next, the cognitive enhancing capacities of TAT-D2pep were estimated in C57BL/6N mice to facilitate working memory in T-maze and cognitive flexibility assessed in LI paradigm disrupted by parametric manipulations using five CS-US pairings.

#### Experiment 3

This experiment probed action of TAT-D2pep to alter synaptic plasticity on hippocampal slices of C57BL/6N mice using electrophysiological approach. To prove dopamine-dependent action of the peptide, D2 receptor agonist (quinpirole) was applied.

## Results

### Experiment 1

#### Efficacy of TAT-D2pep to Rescue the Disrupted Latent Inhibition in Disc1-L100P Mutant Mice

Disc1-L100P mice express deficit of LI, which was rescued by atypical APD, clozapine and pharmacological compounds—GSK-3 and PDE4 inhibitors, as potentially new APDs (Clapcote et al., 2007; Lipina et al., 2011a). TAT-D2pep elicited APD-like effects on PPI and hyperactivity in rodents as it was previously showed (Su et al., 2014). However, the disrupted PPI was also observed in patients with other mental disorders (Braff et al., 2001), whereas impaired LI was reported specifically in patients with schizophrenia (Weiner et al., 1984, 1988; Gray et al., 1992; Thornton et al., 1996; Rascle et al., 2001). Hence, to further prove APD-like activity of TAT-D2pep, the current experiment estimated ability of TAT-D2pep to correct deficit of LI induced by Disc1-L100P mutation.

MANOVA found a main effect of pre-exposure (*F*_(1,51)_ = 62.9; *p* < 0.001), peptide (*F*_(1,51)_ = 11.7; *p* < 0.01), time interval (*F*_(7,357)_ = 105.6; *p* < 0.001), genotype × time interval (*F*_(7,357)_ = 2.6; *p* < 0.05), peptide × pre-exposure (*F*_(1,51)_ = 22.1; *p* < 0.001), pre-exposure × time interval (*F*_(7,357)_ = 25.1; *p* < 0.001), genotype × peptide × pre-exposure (*F*_(1,357)_ = 9.1; *p* < 0.01), genotype × peptide × time interval (*F*_(7,357)_ = 2.1; *p* < 0.05), peptide × pre-exposure × time interval (*F*_(7,357)_ = 7.3; *p* < 0.001) and genotype × peptide × pre-exposure × time interval (*F*_(7,357)_ = 6.2; *p* < 0.001) interactions on percent of freezing. NPE-WT mice expressed significantly higher freezing in response to the CS than PE-WT mice regardless TAT-D2pep-sc TAT-D2-pep treatments (Figures 1A,B). In opposite, both PE and NPE groups of Disc1-L100P mice treated by TAT-D2pep-sc showed comparable freezing in response to the tone (Figure 1C), indicating on their disrupted LI. TAT-D2pep-treated PE-Disc1-L100P mice reduced their freezing, whereas TAT-D2pep-treated NPE Disc1-L100P mice increased freezing as compared to TAT-D2pep-sc-treated Disc1-L100P mice (Figures 1D,E). MANOVA detected a main effect of pre-exposure (*F*_(1,51)_ = 71.3; *p* < 0.001), peptide (*F*_(1,51)_ = 16.6; *p* < 0.001), pre-exposure × peptide (*F*_(1,51)_ = 23.5; *p* < 0.001) and genotype × pre-exposure × peptide (*F*_(1,51)_ = 6.7; *p* < 0.05) on percentage of the averaged freezing (Figure 1E). As can be seen, TAT-D2pep-sc-treated Disc1-L100P mice showed no LI, whereas TAT-D2pep-treated mutant mice expressed robust LI (Figures 1D,E).

### Experiment 2

#### Facilitation of Citive Flexibility Assessed in LI Paradigm by TAT-D2pep in C57BL/6N Mice

Given that APDs were able to correct the impaired LI, induced by extended conditioning in C57BL/6N mice (Lipina et al., 2005; Lipina and Roder, 2010), the current experiment estimated efficacy of TAT-D2pep to facilitate deficit of LI induced by five CS-US as potentially new APD.

*LI with* *two CS-US pairs*: ANOVA found a main effect of pre-exposure (*F*_(1,10)_ = 21.5; *p* < 0.001) on average freezing assessed during 5 min in response to the tone (CS). NPE mice froze significantly more in response to CS (averaged freezing in response to CS: 47.6 ± 8.2%) than PE group (8.6 ± 1.5%; *p* < 0.001). Baseline freezing of C57BL/6N mice of both PE and NPE groups did not differ from each other (*p* > 0.05).

*LI with* *five CS-US pairs*: MANOVA found a main effect of pre-exposure (*F*_(1,20)_ = 9.1; *p* < 0.01), peptide (*F*_(1,20)_ = 5.7; *p* < 0.05) and pre-exposure × peptide interactions (*F*_(1,20)_ = 5.1; *p* < 0.05) on freezing of experimental mice. MANOVA with repeated measures detected a significant effect of time intervals (*F*_(7,140)_ = 59.9; *p* < 0.001), time intervals × peptide (*F*_(7,140)_ = 2.8; *p* < 0.001), time intervals × pre-exposure (*F*_(7,140)_ = 4.1; *p* < 0.001) on freezing behavior. As can be seen in Figure 2A both PE and NPE groups of TAT-D2pep-sc-treated C57BL/6N mice expressed similar amount of freezing in response to CS, which indicates that five CS-US pairs disrupted LI in mice as expected. However, TAT-D2pep-treated PE mice exhibited reduced freezing in response to tone as compared to NPE TAT-D2pep-treated group under the same conditions with 5CS-US training (*p*’s < 0.05–0.01). Hence, TAT-D2pep-treated C57BL/6N mice demonstrated LI (*p*’s < 0.05–0.01; Figures 2B,C).

### Experiment 3

#### Facilitation of Working Memory Assessed in T-Maze by TAT-D2pep in C57BL/6N Mice

In agreement with CNTRICS’ goals (Barak and Weiner, 2011), we probed TAT-D2pep’ capacity to act as a cognitive enhancer and hence, tested its efficacy to facilitate working memory in C57BL/6N mice.

ANOVA revealed a main effect of the peptide (*F*_(1,14)_ = 4.5; *p* < 0.05) on the sequences of visited arms, where TAT-D2pep facilitated the performance of C57BL/6N mice in comparison to TAT-D2sc-treated mice. Figure 2D depicts the percentage of spontaneous alterations in T-maze task. Notably, TAT-D2-pep did not affect motor activity since the number of entries into each arm of T-maze was comparable in mice of both experimental groups (TAT-D2pep-sc: 16.0 ± 1.2 vs. TAT-D2pep: 16.1 ± 1.5).

### Experiment 4

#### Electrophysiological Effects of TAT-D2pep on Synaptic Plasticity in Hippocampal Slices

The unique capacity of TAT-D2pep to rescue the disrupted LI in genetic model (Disc1-L100P mouse line) and induced by parametric manipulations (5CS-US in C57BL/6N inbred strain), as well as to facilitate working memory in C57BL/6N mice likely involves underlying synaptic plasticity. The most direct mechanisms by which TAT-D2pep may regulate cognitive functions is synaptic LTP. Hence, the current experiment assessed ability of TAT-D2pep to facilitate LTP of hippocampal neurons under basal condition and under stimulation of D2 receptors by quinpirole to experimentally prove action of TAT-D2pep via D2 receptors.

Effects of TAT-D2pep on hippocampal synaptic plasticity were studied using LTP as a model of synaptic plasticity in the CA1 pyramidal neurons. TAT-D2pep (10 μM) was added to incubation medium and parameters of fEPSP were measured for 10 min after addition. We did not observe any significant changes in the latency or amplitude of fEPSP (Figures 3D,E). For HFS-induced LTP, TAT-D2pep did not affect the amplitude of fEPSP measured 60 min after HFS compared to that in the control slices (Figure 3H).

Addition of selective D2R agonist quinpirole (10 μM) to incubation medium 10 min prior to HFS did not affect fEPSP (Figures 3F,G), but inhibited LTP expression (Figure 3I). However, 10 min pre-incubation of slices with 10 μM TAT-D2pep abolished this effect of quinpirole (Figure 3I). The fEPSP normalized to baseline measured 60 min after HFS was 1.486 ± 0.108, which was not differ from control (saline application; *P* > 0.05) but significantly differed from other experimental groups (*p*’s < 0.05). TAT-D2pep-sc did not affect the basic parameters fEPSP of CA1 pyramidal neurons (Figures 3B,C). We did not observe significant differences in amplitude and latency of fEPSP after 10 min of incubation with 10 μM TAT-D2pep-sc (Figure 3H). HFS induced LTP in slices incubated with TAT-D2pep-sc and normalized fEPSP amplitude 60 min after HFS in these slices was comparable with control values (Figure 3H). In opposite to TAT-D2pep, TAT-D2pep-sc was unable to reverse the quinpirole-induced inhibition of LTP (Figure 3I).

## Discussion

The results of the present study can be summarized as follows: (1) Deficit of LI in Disc1-L100P mutant mice with facilitated DISC1 × D2R interactions was corrected by TAT-D2pep uncoupling peptide. (2) Uncoupling DISC1 × D2R protein-protein interactions improved working memory and ameliorated latent inhibition disrupted by parametric manipulations in C57BL/6N inbred mice. (3) TAT-D2pep did not affect LTP under basal conditions, but reversed the deficit in LTP induced by quinpirole-induced D2R stimulation. Overall, we found that TAT-D2pep facilitated synaptic plasticity upon D2R stimulation, which may contribute to its action as APD with nootropic capacity.

Our recent discovery of DISC1xD2R protein-protein interaction implicated DISC1 as a new molecular regulator of D2R availability on the cellular membrane, acting via GSK-3-dependent D2R internalization mechanisms upon D2R stimulation (Su et al., 2014). Uncoupling of the facilitated DISC1xD2R interaction induced by Disc1-L100P mutation in a mouse by the peptide induced antipsychotic effects on genetic (Disc1-L100P mutant line) and pharmacological (amphetamine) models of schizophrenia (Su et al., 2014). Our current results demonstrated that TAT-D2pep was also able to rescue LI deficit in Disc1-L100P mice, behind hyperactivity and PPI deficit previously reported by us (Su et al., 2014). Hence, DISC1xD2R protein-protein interaction may offer a new therapeutic target in the field of psychopharmacology.

Cognitive impairments in patients with schizophrenia are most resistant to treatment by commonly used APDs. Hence, the lack of efficacy of APDs switched the direction of drug discovery towards generation of new APDs with capacities as cognitive enhancers. Nootropic drugs were studied for a long time to improve such cognitive functions as attention, motivational aspect, learning and memory in several mental disorders (Lanni et al., 2008). These cognitive enhancers, include compounds with various mechanisms of action, such as vitamins/supplements (vitamins B, D, omega-3), racetams (piracetam, oxiracetam), stimulants (amphetamine, nicotine) and dopaminergic drugs. However, although a wide range of nootropic compounds have been tested, only a few of them were able to improve cognitive symptoms in patients with schizophrenia: e.g., inhibitors of glycine transporter-1 (GlyT1), GABA(A)α5 inverse agonists (reviewed Wallace et al., 2011) or amphetamine (Barch and Carter, 2005; Pietrzak et al., 2010). Amphetamine improved speed performance, spatial working memory (Pietrzak et al., 2010), language production, executive function, visual attention and vigilance in patients with schizophrenia (Barch and Carter, 2005; Pietrzak et al., 2010). GlyT1 inhibitor, sarcosine, ameliorated cognitive symptoms in schizophrenics when added to APD, risperidone but not clozapine (Tsai et al., 1998; Lane et al., 2006). Similarly, d-serine (agonist of the glycine site of NMDA receptors) improved executive function in patients with schizophrenia co-administrated with APDs (Goff et al., 1995), although d-cycloserine alone had limited efficacy in this regard (Otto et al., 2009).

As a first step to explore behavioral effects of TAT-D2pep as a cognitive enhancer based on recommendations of CNTRICS (Barch et al., 2009), we have probed its effects on working memory and cognitive flexibility, as schizophrenia-related cognitive phenotypes, assessed in T-maze and LI tests in control C57BL/6N inbred mice. It still remains to be explored further if TAT-D2pep may affect various cognitive domains, including e.g., executive functions, episodic memory, spatial learning and memory or motivational aspects to identify its specificity and efficacy in the future studies. Nevertheless, there is a need to understand how potentially new cognitive enhancers will act on normal individuals before testing such compounds in patients with schizophrenia to correct their cognitive deficits. So, typical APDs, sulpiride or clozapine were able to improve attentional deficit and impaired working memory in rodents with disrupted prefrontal cortex but these APDs impaired cognitive performance in control animals (Murphy et al., 1997; Passetti et al., 2003a,b; Baviera et al., 2008). Another example is that the chronic treatment with clozapine improved deficit of working memory induced by maternal immune activation, but negatively affected working memory in mice born to control mothers (Meyer et al., 2010).

Acute administration of TAT-D2pep improved percentage of spontaneous alterations in C57BL/6N mice without changes of their motor activity, suggesting that it mainly acts on working memory. Interestingly, similar effect on working memory has been obtained after administration of dual inhibitor of GSK-3 and PDE7—VP1.15 (Lipina et al., 2013), GSK-3 inhibitor (VP3.36; Lipina et al., 2016) as well as in PDE4B-Y358C mutant mouse line (McGirr et al., 2016), supporting the contribution of DISC1 interactome in cognitive enhancement.

Patients with schizophrenia also have impaired attention, including an inability to ignore irrelevant stimuli (Heinrichs and Zakzanis, 1998; Morris et al., 2013). LI reflects a process of learning to ignore irrelevant stimulus (CS) and has a long history in clinical and animal studies (Lubow, 2010). In other words, LI is impaired learning of CS-US associations in PE group, which was pre-exposed to CS without reinforcement in comparison with NPE group, without pre-exposures to the CS. APDs in humans and animals potentiate disrupted LI (Moser et al., 2000; Lipina et al., 2005, 2011b; Weiner and Arad, 2009; Lipina and Roder, 2010). To further explore the possibility of TAT-D2pep as a new APD, we probed effects of the peptide on the facilitation of LI disrupted by parametric manipulation. First, LI phenomenon was observed in C57BL/6N mice with 40 pre-exposures and two CS-US conditioning trials, which is in agreement with previous study (Rimer et al., 2005). However, five CS-US pairings were able to disrupt LI in mice, supporting our previous report (Lipina et al., 2005). TAT-D2pep facilitated the disrupted LI in mice, supporting further its APD-like activity reported earlier (Su et al., 2014), selectively reducing freezing in response to the conditioned stimulus in the PE group of mice, i.e., decreasing their capacity to “switch” attention once CS, as previously irrelevant stimulus, become relevant. Notably, TAT-D2pep had no effect of fear memory in the NPE group as freezing levels in NPE TAT-D2pep- and TAT-D2pep-sc-treated mice were comparable. Several compounds were able to facilitate the disrupted LI by parametric manipulations eliciting APD-like activity in rats (Weiner and Feldon, 1997) and mice (Lipina et al., 2005; Lipina and Roder, 2010; Lipina et al., 2005, 2013), including clozapine, d-serine, GlyT1 inhibitor (ALX5407; Lipina et al., 2005), rolipram and haloperidol (Lipina and Roder, 2010), GSK-3 blocker (TDZD-8; Lipina et al., 2011a) and dual inhibitor of GSK-3 and PDE7—VP1.15 (Lipina et al., 2013). This effect is specific for APDs and is not produced by a wide range of non-APDs (Dunn et al., 1993). Interestingly, clozapine inhibits GSK-3 activity in cell culture (Aubry et al., 2009), the rat frontal cortex (Roh et al., 2007) and the mouse brain (Li et al., 2007), suggesting that GSK-3 underlies APD-like activity. Study of the molecular mechanisms of TAT-D2pep action (Su et al., 2014) revealed that the peptide corrected the reduced phosphorylation of GSK-3, reflecting its increased enzymatic activity, induced by quinpirole D2R agonist (Su et al., 2014). Moreover, TAT-D2pep facilitates β-arrestin-2 × clathrin complex formation, promoting D2R internalization and hence, reducing its availability on cell surface (Su et al., 2014).

Next, we asked whether uncoupling Disc1 × D2R protein-protein interactions by the TAT-D2pep is able to alter synaptic plasticity, which may underlie its nootropic and APDs activities. First, we did not find any significant facilitation of LTP by TAT-D2pep in control C57BL/6N mice. This finding could be viewed as added bonus of TAT-D2pep since acute administration of APDs impaired LTP in WT animals in the majority of studies (reviewed in Price et al., 2014). LTP is generally regarded as the cellular basis of neuroplasticity which underlies learning and long-term memory (Malenka and Bear, 2004). We found that activation of D2 receptors by quinpirole significantly abolished hippocampal LTP, in a way similar to the methamphetamine-induced LTP impairment (Ishikawa et al., 2005), which was reversed by TAT-D2pep. This observation supports the efficacy of the peptide to reverse amphetamine-induced hyperactivity and apomorphine-induced PPI deficit in rats (Su et al., 2014). Whether the coincident of TAT-D2pep on behavior and synaptic plasticity is caused by causality between these two processes remains to be explored. Nevertheless, we suggest that exploration of novel environment (in our case T-maze) *per se* increases dopamine release in the hippocampus (Moreno-Castilla et al., 2017) which may be mimicked to some extend by quinpirole and, hence, the subsequent increase in D2R internalization induced by TAT-D2pep might stabilize dopamine neurotransmission and as result facilitate LTP and improve working memory in mice. To our knowledge there is no direct evidence for such association, although recent study demonstrated link between DAT internalization and LTP facilitation, induced by environmental enrichment (Kim et al., 2016).

## Conclusion

In summary, our current study provided further evidence that TAT-D2pep uncoupling DISC1 × D2R interactions elicited ability as antipsychotic drug in LI paradigm in genetic mouse model of schizophrenia—Disc1-L100P mice. Moreover, our new findings demonstrated for the first time, that the peptide that uncouples the DISC1 × D2R protein-protein interactions also exhibited effects as a cognitive enhancer by facilitating of working memory and cognitive flexibility in C57BL/6N mice and was able to corrected LTP deficits induced by D2R stimulation. Future studies should focus on impact of balanced DISC1 × D2R protein-protein interactions and the precise molecular/cellular mechanisms of TAT-D2pep action, with the aim to improve diagnosis and treatment of schizophrenia.

## Author Contributions

TL designed the experiments, analyzed data and wrote the manuscript. NB conducted electrophysiological experiments, analyzed data and wrote the manuscript. AT performed behavioral experiments and analyzed data. EP analyzed data. MS analyzed data and wrote the manuscript. QZ and SL wrote the manuscript.

## Conflict of Interest Statement

The authors declare that the research was conducted in the absence of any commercial or financial relationships that could be construed as a potential conflict of interest.
